# Editorial: The intricate web of gastrointestinal virome, mycome and archaeome: implications for gastrointestinal diseases

**DOI:** 10.3389/fgene.2024.1463350

**Published:** 2024-08-09

**Authors:** Asrar Ahmad, Mohammad Ashfaq, Mudasir Rashid

**Affiliations:** ^1^ Center for Sickle Cell Disease, Howard University, Washington, DC, United States; ^2^ The University Centre for Research and Development (UCRD), Chandigarh University, Mohali, Punjab, India; ^3^ Department of Medicine and Cancer Center, Howard University, Washington, DC, United States

**Keywords:** virome, mycome, archaeome, human health and disease, gastroinestinal tract

The gastrointestinal (GI) tract is a complex ecosystem containing bacteria, viruses (virome), fungi (mycome), and archaea (archaeome). It plays a significant role in ecological functioning, evolutionary changes, and host-microbiome interactions (Ogilvie and Jones, 2017; Daliri et al., 2020; Piewngam et al., 2020; Vemuri et al., 2020). Recent advances in sequencing technologies and metagenomic approaches have expanded our comprehension of the gastrointestinal-microbiome axis and its roles in maintaining gut homeostasis, health, and disease (Lepage et al., 2013; Gao et al., 2021; Lau and Yu, 2022). While bacteria have been extensively studied, the virome, mycome, and archaeome have recently gained attention. The human gut virome varies between individuals, composition and dynamics (Minot et al., 2011; Foca et al., 2015). They play a crucial role in gut regulation and contributing to disease development through interactions with the microbiota and immune cells (Ogilvie and Jones, 2017; Santiago-Rodriguez and Hollister, 2019; Hou et al., 2022; Tiamani et al., 2022). The gut mycobiome also plays a critical role in human health and disease, with potential implications for infants, obesity, inflammatory bowel disease (IBD) and neurodegenerative diseases (Forbes et al., 2018; Jain et al., 2021). The archaeome, a diverse and potentially important component of human gut host-associated microbiomes, plays an important role in gut physiology and health, in shaping the health and fitness of the animals (Gaci et al., 2014; Lurie-Weinberger and Gophna, 2015; Borrel et al., 2020; Kim et al., 2020). Studies have shown that Archaea play a key role in the pathogenesis of IBD and the risk of pediatric asthma (Barnett et al., 2019; Singh et al., 2023). However, their role in human health remains poorly understood, therefore, understanding these microbial communities offers new therapeutic approaches for GI disorders and enhances strategies for improving GI health as shown in Figure 1.

**FIGURE 1:**
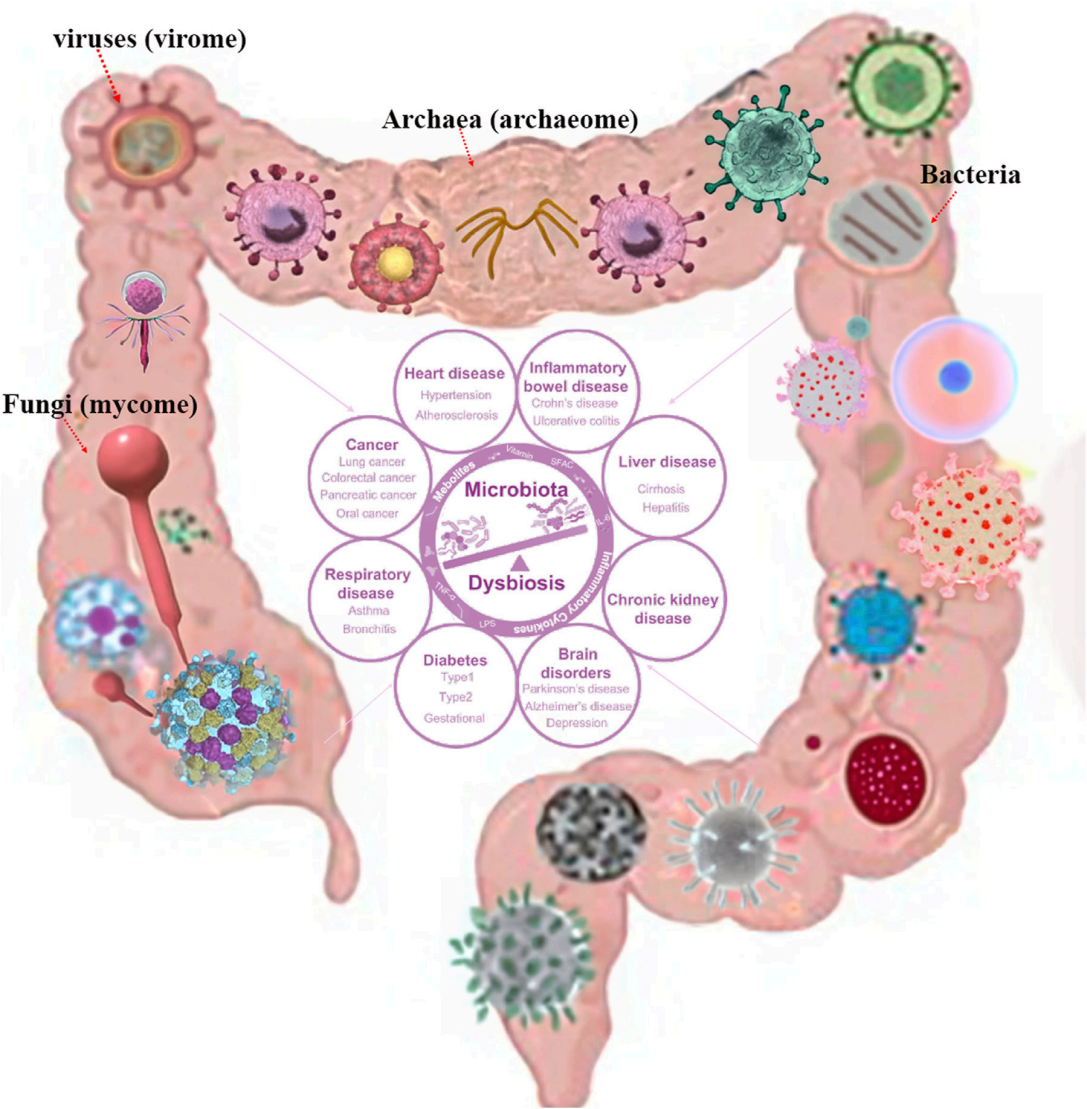
Human microbiota composition with potential consequences. Permission obtained from Hou et al. (2022), Spencer et al. (2022).

The aim of the editorial *“The intricate web of gastrointestinal virome, mycobiome, and archaeome: implications for gastrointestinal diseases”* is to explore the roles and interactions of the gastrointestinal microbiome in relation to gastrointestinal diseases. The objectives include reviewing current research on these microbial communities, discussing their implications for gastrointestinal health, highlighting potential therapeutic implications, and fostering a deeper understanding among healthcare professionals and researchers and suggest avenues for future research and clinical application in this evolving field. Recent studies have shown that, Postherpetic Neuralgia (PHN) is a chronic neuropathic pain syndrome caused by Herpes Zoster virus (HZV) reactivation, and it is more common in older and immunocompromised individuals, and vaccination is effective in preventing complications (Oxman et al., 2005; Mallick-Searle et al., 2016; Ferreira Sampaio et al., 2023). Additionally, it has been shown that HSV patients with peptic ulcer disease may be at a greater risk for developing PHN due to impaired cellular immunity and low nutritional status (Chen et al., 2013). Zhimin et al. have reported that the causal effect between HZV and PHN and the gut microbiota using bidirectional two-sample Mendelian randomization (MR) analysis. The study revealed the alterations in gut microbiota may influence susceptibility to HSV and the development of PHN. This research highlights the potential for specific gut microbiota to serve as a therapeutic target, offering new avenues for the prevention and treatment of viral infections and associated chronic conditions. This study highlights the importance of considering the gut microbiota not just in isolation but as a critical component in the overall immune response and susceptibility to viral infections. By understanding these causal relationships, new therapeutic strategies could be developed, focusing on modulating the gut microbiota to prevent or mitigate the effects of HSV associated PHN. Additionally, Hanjing et al. carried out the study in China (Jinjiang, Fujian), and reported the interactions between *Helicobacter pylori*, chronic gastritis, and the gut microbiota. They demonstrate that how *Helicobacter. pylori* infection and chronic gastritis are intricately linked with changes in gut microbiota composition. These findings emphasize the need for a comprehensive approach in managing *Helicobacter. pylori* infections and gastritis, considering their impact on the overall gut microbiota health. Moreover, gut microbiome-associated metabolites in serum can accurately distinguish colorectal cancer and adenomas from normal samples (Chen et al., 2022). Also, another study profiled the serum antibody responses of 997 healthy individuals against 244,000 peptide antigens derived from gut microbiota, data indicated that the serum antibody repertoires displayed high diversity and stability against human microbiota and identified several factors influencing the serum antibody response, such as age, gender, body mass index, and geographical location. These findings shed light on the role of the immune system in maintaining homeostasis with the gut microbiota (Vogl et al., 2021). Chen et al. present a differential analysis of serum immunology and gut microbiota in patients suffering from various gastrointestinal diseases. The study identifies distinct immunological and microbiota profiles associated with specific GI conditions, providing valuable insights into the interplay between the immune system and gut microbiota. This research highlights the potential for personalized medicine approaches in GI, tailoring treatments to the unique microbiota like recent report has shown that smart antibiotic (Lolamicin) can target a group of harmful microbes but does not disturb those that live peacefully in the gut (Munoz et al., 2024). Furthermore, Xiao et al. examine the causal relationship between the gut microbiome and liver cirrhosis through a bi-directional two-sample Mendelian randomization analysis. The results highlight significant causal links, suggesting that specific alterations in the gut microbiome may contribute to the development of liver cirrhosis. This study points out the importance of considering gut microbiota health in the prevention and management of liver diseases. Also, other evidence has shown that gut microbiome plays a significant role in liver disorders, potentially contributing to disease process and severity. Prebiotics and probiotics may be effective treatments for complications and liver cirrhosis. Additionally, the gut-liver axis plays a crucial role in liver disease, and progressive changes in the gut microbiome accompany cirrhosis. It became more severe in the setting of decompensation, with the cirrhosis dysbiosis ratio being a useful quantitative index (Bajaj et al., 2014; Giannelli et al., 2014; Qin et al., 2014; Tilg et al., 2016; Albillos et al., 2020; Li et al., 2022). This issue opens a new avenue for preventive strategies and therapeutic interventions aimed at modulating the gut microbiome to improve health and patient quality of life.

## Conclusion

The intricate interplay between the gut microbiota, virome, mycome, and archaeome has profound implications for GI diseases and these studies provide a foundation for future research and potential therapeutic innovations. This editorial reveals how these microbial communities influence conditions such as HSV, chronic gastritis, liver cirrhosis and emphasize the potential of targeting gut microbiota for therapeutic interventions. Understanding the intricate web of these microorganisms opens new avenues for personalized medicine, aiming to improve GI health through advanced sequencing technologies and metagenomic analyses (Brim et al., 2017; Liang et al., 2017). We hope this issue inspires further exploration into the intricate web of the GI ecosystem and its impact on health, fostering a deeper understanding that can translate into better clinical practices and holds vast potential for unlocking new paradigms in the treatment and management of gastrointestinal diseases to improve patient outcomes.

## References

[B1] Albillos A. de Gottardi A. Rescigno M. (2020). The gut-liver axis in liver disease: pathophysiological basis for therapy. J. Hepatol. 72, 558–577. 10.1016/j.jhep.2019.10.003 31622696

[B2] Bajaj J. S. Heuman D. M. Hylemon P. B. Sanyal A. J. White M. B. Monteith P. (2014). Altered profile of human gut microbiome is associated with cirrhosis and its complications. J. Hepatol. 60, 940–947. 10.1016/j.jhep.2013.12.019 24374295 PMC3995845

[B3] Barnett D. J. M. Mommers M. Penders J. Arts I. C. W. Thijs C. (2019). Intestinal archaea inversely associated with childhood asthma. J. Allergy Clin. Immunol. 143, 2305–2307. 10.1016/j.jaci.2019.02.009 30796982

[B4] Borrel G. Brugere J. F. Gribaldo S. Schmitz R. A. Moissl-Eichinger C. (2020). The host-associated archaeome. Nat. Rev. Microbiol. 18, 622–636. 10.1038/s41579-020-0407-y 32690877

[B5] Brim H. Yooseph S. Lee E. Sherif Z. A. Abbas M. Laiyemo A. O. (2017). A microbiomic analysis in african Americans with colonic lesions reveals Streptococcus sp.VT162 as a marker of neoplastic transformation. Genes (Basel) 8, 314. 10.3390/genes8110314 29120399 PMC5704227

[B6] Chen F. Dai X. Zhou C. C. Li K. X. Zhang Y. J. Lou X. Y. (2022). Integrated analysis of the faecal metagenome and serum metabolome reveals the role of gut microbiome-associated metabolites in the detection of colorectal cancer and adenoma. Gut 71, 1315–1325. 10.1136/gutjnl-2020-323476 34462336 PMC9185821

[B7] Chen J. Y. Chang C. Y. Lan K. M. Sheu M. J. Lu C. L. Hu M. L. (2013). Is peptic ulcer disease a risk factor of postherpetic neuralgia in patients with herpes zoster? Med. Hypotheses 81, 834–838. 10.1016/j.mehy.2013.09.007 24074834

[B8] Daliri E. B. Ofosu F. K. Chelliah R. Lee B. H. Oh D. H. (2020). Health impact and therapeutic manipulation of the gut microbiome. High. Throughput 9, 17. 10.3390/ht9030017 32751130 PMC7564083

[B9] Ferreira Sampaio R. Vinicius da Silva Pereira M. Nayara Coelho Muniz Cardoso M. Soares da Costa D. Saraiva Sales E. Vinicius Magalhães Guedes M. (2023). Integrative literature review: prevention of neurological complications of herpes zoster in adults - advances, challenges and perspectives. Health. Soc. 3, 16–30. 10.51249/hs.v3i04.1447

[B10] Foca A. Liberto M. C. Quirino A. Marascio N. Zicca E. Pavia G. (2015). Gut inflammation and immunity: what is the role of the human gut virome? Mediat. Inflamm. 2015, 326032. 10.1155/2015/326032 PMC4405218 25944980

[B11] Forbes J. D. Bernstein C. N. Tremlett H. Van Domselaar G. Knox N. C. (2018). A fungal world: could the gut mycobiome Be involved in neurological disease? Front. Microbiol. 9, 3249. 10.3389/fmicb.2018.03249 30687254 PMC6333682

[B12] Gaci N. Borrel G. Tottey W. O'Toole P. W. Brugere J. F. (2014). Archaea and the human gut: new beginning of an old story. World J. Gastroenterol. 20, 16062–16078. 10.3748/wjg.v20.i43.16062 25473158 PMC4239492

[B13] Gao B. Chi L. Zhu Y. Shi X. Tu P. Li B. (2021). An introduction to next generation sequencing bioinformatic analysis in gut microbiome studies. Biomolecules 11, 530. 10.3390/biom11040530 33918473 PMC8066849

[B14] Giannelli V. Di Gregorio V. Iebba V. Giusto M. Schippa S. Merli M. (2014). Microbiota and the gut-liver axis: bacterial translocation, inflammation and infection in cirrhosis. World J. Gastroenterol. 20, 16795–16810. 10.3748/wjg.v20.i45.16795 25492994 PMC4258550

[B15] Hou K. Wu Z. X. Chen X. Y. Wang J. Q. Zhang D. Xiao C. (2022). Microbiota in health and diseases. Signal Transduct. Target Ther. 7, 135. 10.1038/s41392-022-00974-4 35461318 PMC9034083

[B16] Jain U. Ver Heul A. M. Xiong S. Gregory M. H. Demers E. G. Kern J. T. (2021). Debaryomyces is enriched in Crohn's disease intestinal tissue and impairs healing in mice. Science 371, 1154–1159. 10.1126/science.abd0919 33707263 PMC10114606

[B17] Kim J. Y. Whon T. W. Lim M. Y. Kim Y. B. Kim N. Kwon M. S. (2020). The human gut archaeome: identification of diverse haloarchaea in Korean subjects. Microbiome 8, 114. 10.1186/s40168-020-00894-x 32753050 PMC7409454

[B18] Lau H. C. H. Yu J. (2022). Uncovering novel human gut virome using ultra-deep metagenomic sequencing. Chin. Med. J. Engl. 135, 2395–2397. 10.1097/CM9.0000000000002382 36583859 PMC9945418

[B19] Lepage P. Leclerc M. C. Joossens M. Mondot S. Blottiere H. M. Raes J. (2013). A metagenomic insight into our gut's microbiome. Gut 62, 146–158. 10.1136/gutjnl-2011-301805 22525886

[B20] Li R. Yi X. Yang J. Zhu Z. Wang Y. Liu X. (2022). Gut microbiome signatures in the progression of hepatitis B virus-induced liver disease. Front. Microbiol. 13, 916061. 10.3389/fmicb.2022.916061 35733959 PMC9208012

[B21] Liang Q. Chiu J. Chen Y. Huang Y. Higashimori A. Fang J. (2017). Fecal bacteria act as novel biomarkers for noninvasive diagnosis of colorectal cancer. Clin. Cancer Res. 23, 2061–2070. 10.1158/1078-0432.CCR-16-1599 27697996

[B22] Lurie-Weinberger M. N. Gophna U. (2015). Archaea in and on the human body: health implications and future directions. PLoS Pathog. 11, e1004833. 10.1371/journal.ppat.1004833 26066650 PMC4466265

[B23] Mallick-Searle T. Snodgrass B. Brant J. M. (2016). Postherpetic neuralgia: epidemiology, pathophysiology, and pain management pharmacology. J. Multidiscip. Healthc. 9, 447–454. 10.2147/JMDH.S106340 27703368 PMC5036669

[B24] Minot S. Sinha R. Chen J. Li H. Keilbaugh S. A. Wu G. D. (2011). The human gut virome: inter-individual variation and dynamic response to diet. Genome Res. 21, 1616–1625. 10.1101/gr.122705.111 21880779 PMC3202279

[B25] Munoz K. A. Ulrich R. J. Vasan A. K. Sinclair M. Wen P. C. Holmes J. R. (2024). A Gram-negative-selective antibiotic that spares the gut microbiome. Nature 630, 429–436. 10.1038/s41586-024-07502-0 38811738 PMC12135874

[B26] Ogilvie L. A. Jones B. V. (2017). The human gut virome: form and function. Emerg. Top. Life Sci. 1, 351–362. 10.1042/ETLS20170039 33525769

[B27] Oxman M. N. Levin M. J. Johnson G. R. Schmader K. E. Straus S. E. Gelb L. D. (2005). A vaccine to prevent herpes zoster and postherpetic neuralgia in older adults. N. Engl. J. Med. 352, 2271–2284. 10.1056/NEJMoa051016 15930418

[B28] Piewngam P. De Mets F. Otto M. (2020). Intestinal microbiota: the hidden gems in the gut? Asian Pac J. Allergy Immunol. 38, 215–224. 10.12932/AP-020720-0897 33068364 PMC10988648

[B29] Qin N. Yang F. Li A. Prifti E. Chen Y. Shao L. (2014). Alterations of the human gut microbiome in liver cirrhosis. Nature 513, 59–64. 10.1038/nature13568 25079328

[B30] Santiago-Rodriguez T. M. Hollister E. B. (2019). Human virome and disease: high-throughput sequencing for virus discovery, identification of phage-bacteria dysbiosis and development of therapeutic approaches with emphasis on the human gut. Viruses 11, 656. 10.3390/v11070656 31323792 PMC6669467

[B31] Singh P. Murugesan S. Kumar M. Saadaoui M. Elhag D. A. Hendaus M. A. (2023). P915 Uncovering the role of Archaea in the pathogenesis of Inflammatory bowel disease. J. Crohn's Colitis 17, i1026. 10.1093/ecco-jcc/jjac190.1045

[B36] Spencer L. Olawuni B. Singh P. (2022). Gut virome: role and distribution in health and gastrointestinal diseases. Front. cell. infect. microbiol. 12, 836706. 10.3389/fcimb.2022.836706 35360104 PMC8960297

[B32] Tiamani K. Luo S. Schulz S. Xue J. Costa R. Khan Mirzaei M. (2022). The role of virome in the gastrointestinal tract and beyond. FEMS Microbiol. Rev. 46, fuac027. 10.1093/femsre/fuac027 35700129 PMC9629487

[B33] Tilg H. Cani P. D. Mayer E. A. (2016). Gut microbiome and liver diseases. Gut 65, 2035–2044. 10.1136/gutjnl-2016-312729 27802157

[B34] Vemuri R. Shankar E. M. Chieppa M. Eri R. Kavanagh K. (2020). Beyond just bacteria: functional biomes in the gut ecosystem including virome, mycobiome, archaeome and helminths. Microorganisms 8, 483. 10.3390/microorganisms8040483 32231141 PMC7232386

[B35] Vogl T. Klompus S. Leviatan S. Kalka I. N. Weinberger A. Wijmenga C. (2021). Population-wide diversity and stability of serum antibody epitope repertoires against human microbiota. Nat. Med. 27, 1442–1450. 10.1038/s41591-021-01409-3 34282338

